# A Review of Methods for Removal of Ceftriaxone from Wastewater

**DOI:** 10.3390/jox12030017

**Published:** 2022-08-02

**Authors:** Petro Karungamye, Anita Rugaika, Kelvin Mtei, Revocatus Machunda

**Affiliations:** 1 Department of Chemistry, The University of Dodoma (UDOM), Dodoma P.O. Box 338, Tanzania; 2 School of Materials Energy Water and Environmental Sciences, The Nelson Mandela African Institution of Science and Technology, Arusha P.O. Box 447, Tanzania; anita.rugaika@nm-aist.ac.tz (A.R.); kelvin.mtei@nm-aist.ac.tz (K.M.); revocatus.machunda@nm-aist.ac.tz (R.M.)

**Keywords:** antibiotics, ceftriaxone, wastewater treatment, degradation, removal

## Abstract

The presence of pharmaceuticals in surface water and wastewater poses a threat to public health and has significant effects on the ecosystem. Since most wastewater treatment plants are ineffective at removing molecules efficiently, some pharmaceuticals enter aquatic ecosystems, thus creating issues such as antibiotic resistance and toxicity. This review summarizes the methods used for the removal of ceftriaxone antibiotics from aquatic environments. Ceftriaxone is one of the most commonly prescribed antibiotics in many countries, including Tanzania. Ceftriaxone has been reported to be less or not degraded in traditional wastewater treatment of domestic sewage. This has piqued the interest of researchers in the monitoring and removal of ceftriaxone from wastewater. Its removal from aqueous systems has been studied using a variety of methods which include physical, biological, and chemical processes. As a result, information about ceftriaxone has been gathered from many sources with the searched themes being ceftriaxone in wastewater, ceftriaxone analysis, and ceftriaxone removal or degradation. The methods studied have been highlighted and the opportunities for future research have been described.

## 1. Introduction

**Figure 1:**
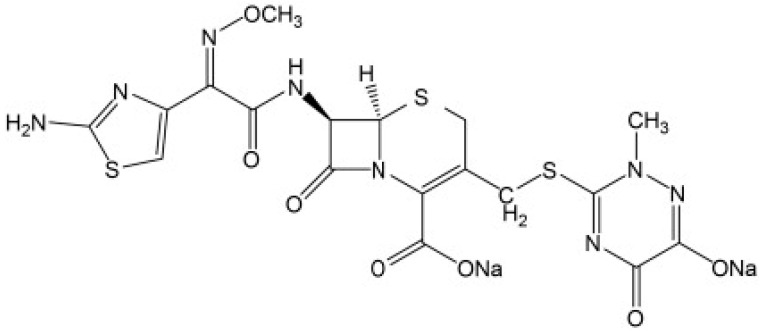
Chemical structure of ceftriaxone [17].

Pharmaceutical traces, and their metabolites and degradation products have been found in both surface and ground water across the globe [1]. Antibiotics contribute a higher proportion to this in pharmaceutical wastewater [2] due to their significant use [3]. They are used to treat different diseases and bacterial infections in human beings and other animals [4,5,6]. Wastewater containing such complex components becomes difficult to treat [7,8]. Their high solubility in aqueous systems, longer half-life [9], and low biodegradability [10] makes them accumulate over time. Ceftriaxone (refer Figure 1) is a type of antibiotic used to treat a variety of bacterial illnesses. It is a 3rd generation cephalosporin that inhibits the formation of mucopeptide in bacterial cell walls [11]. Its systematic chemical name is [6R-[6a,7b,(Z)]]-5-thia-1-azabicclo-[4.2.0]-oct-2-ene-2-carboxylicacid,7-[[(2-amino-4-thiazolyl)(methoxyimino)-acetyl]amino]-8-oxo-3-[[(1,2,5,6-tetrahydro-2-methyl-5,6-dioxo-1-2,4–triazin-3-yl)-thio]methyl]]-, disodium salt [12]. It is widely used in clinical settings due to its strong antibacterial effect, good lactamase tolerance, good clinical effect, low toxicity, and low allergic reaction [13].

Like other 3rd generation cephalosporins, this antibiotic is less effective against Gram-positive bacteria compared to first-generation medicines, but it has a far larger spectrum of activity against Gram-negative bacteria [14]. Ceftriaxone has been useful for the treatment of infections caused by susceptible organisms in the lower respiratory tract, abdomen, skin and soft tissue, pelvic area, bone and joint, meninges, and urinary tract [15]. Based on intramuscular injections, ceftriaxone is 100% bioavailable and it is removed by biliary and renal excretion [16].

The antibiotics used for animals and humans’ treatment enter the environment via urine and feces, thus optimizing and/or limiting antibiotic use, which is essential to minimize contamination of the environment [18]. It is believed that around 40–90% of the prescribed antibiotic dose (depending on the class of pharmaceutical) is excreted as a parent compound in the active form in the feces and urine, and when it finally reaches the environment it causes soil, water, and plant contamination [19,20,21]. The use of excessive doses of antibiotics in livestock farming can pollute agro-ecosystems through either the application of contaminated manure as fertilizer in agriculture, or the irrigation of farms with wastewater [22,23]. Another source of concern comprises the improper disposal of leftover, expired, or unused pharmaceuticals which are released into sewage systems [24]. Due to the incomplete removal of pharmaceutical compounds and their metabolites by conventional treatment technologies, several pharmaceutical residues have been detected in wastewater effluents. This makes antibiotics present in wastewater treatment plants sludge and, finally, effluent [25,26,27]. Hospital effluents also comprise a significant source of antibiotics and antibiotic-resistant microorganisms in the environment [28].

Although antibiotic residue quantities in aquatic environments range from ng/L to µg/L, their continual discharge and persistence may have unexpected consequences for non-target aquatic organisms [11]. Antibiotics in water resources can generate a wide range of issues, including toxicity on aquatic organisms such as bacteria, algae, crabs, and fish, and increased antibiotic resistance in bacteria [5,29]. According to the WHO [23], antibiotic resistance is one of the three biggest dangers to human health. As a result, developing effective and environmentally friendly methods to break down those antibiotics in the aquatic environment is critical [2,4,30]. Being an antibiotic, ceftriaxone has similar effects. Due to its widespread usage in medicine and veterinary medicine, ceftriaxone contributes significantly to environmental pollution [9].

Several studies show that ceftriaxone aqueous solution is unstable, with a stability that is pH and temperature-dependent. The ideal pH for ceftriaxone stability in aqueous solution is 7.5, and when maintaining this pH for more than 6 h at 37 °C, only around 10% of ceftriaxone can be degraded. However, degradation occurs more quickly at lower or higher pH levels. The aqueous solution of ceftriaxone is stable for 4 days at room temperature in the presence and absence of light, and that ceftriaxone is stable for a longer amount of time at lower temperatures, but it decomposes after a specific period of time [31]. More characteristics of ceftriaxone are presented in Table 1.

Ceftriaxone in wastewater has been reported by various researchers. For instance, research was conducted in India to examine the effluent of selected health care establishments and municipal wastewater treatment plants. The study findings indicated that the results for ceftriaxone ranged from 1.25–29.15 µg/mL [32]. The antibiotics have been proven in several publications as emergent contaminants in the aquatic environments around the world. However, the majority of the findings are from outside Africa [33]. Therefore, the purpose of this literature review was to analyze the information available in relation to the techniques for the removal of ceftriaxone from wastewater systems. The reviewed literature employed electronic databases, manual searches of reference lists from chosen electronic publications, and internet search engines to find relevant literature on the occurrence, concentrations, and techniques used to examine ceftriaxone in wastewater. The expressions ceftriaxone in wastewater, ceftriaxone analysis, and ceftriaxone removal or degradation were searched in Google Scholar, PubMed, Science Direct, Scopus, Taylor & Francis online, Web of Science, and Wiley Online Library. The search was limited to articles written in the English language.

## 2. Methods Used to Analyze Antibiotics

Various methods have been developed to detect and quantify antibiotics in various types of samples. The referred methods include chromatographic, spectrophotometric, and electrochemical methods [34]. High performance liquid chromatography (HPLC) is, by far, the most extensively utilized instrumental method in pharmaceuticals analysis [35].

### 2.1. Chromatographic Methods

Pharmaceuticals and their metabolites have been analyzed using a variety of chromatographic methods. Such methods can be used alone or hyphenated with mass spectrometry. Mass spectrometry-based approaches, particularly liquid chromatography, coupled with tandem mass spectrometry (LC/MS/MS) can reach extraordinarily high degrees of specificity compared with immunoassay or even chromatographic detection utilizing detectors such as UV or fluorescence. The specificity and sensitivity of a chromatographic method are controlled by chromatographic conditions such as choice of mobile phase and analytical column, detector, and sample preparation [36]. For antibiotic analysis, the analytical method is selected based on the characteristics of the analyzed antibiotic, which includes solubility in water and organic solvents or acid-base properties [37].

Thin-layer chromatography (TLC) is one of the most important analytical methods used to determine the qualitative and semiquantitative levels of pharmaceuticals in various types of samples [34]. TLC is usually applied as the quick, easy, and straightforward procedure. The effective separation is determined by the sample’s properties as well as the properties of the stationary and mobile phases [38]. TLC can successfully be used for preliminary screening of the pharmaceutical compounds. It is commonly employed in contemporary analysis as a separation method to determine the presence or absence of antibiotics over a predetermined concentration level [38,39]. It can also be used to evaluate and categorize pure and impure antibiotic preparations as well as assay antibiotics quantitatively in bulk or pharmaceutical preparations [40,41].

Gas chromatography (GC) is a commonly used analytical technique that combines separation chromatographic stage with measurement capacity. GC employs the gas as the mobile phase and coating inside the long capillary column or, less typically, the tiny particles of a solid material packed in a column as the stationary phase. The sample in GC should be able to evaporate so that it flows with the gaseous mobile phase. The temperature gradient to which the chromatographic column is subjected is frequently utilized to speed up the elution of less volatile substances that would otherwise take a long time to elute. The detector signals for the sample’s eluting components are used for quantitative and qualitative analysis [42]. GC is a useful technique for evaluating pharmaceutically relevant substances [43] and impurities [44]. Many pharmaceutical chemicals, however, cannot be gas chromatographed in their natural state and must be transformed into stable and volatile derivatives in order to accomplish successful GC elution and separation. The derivatives are sometimes created in order to attain the appropriate sensitivity, selectivity, or specificity for a given separation [44,45].

High-performance liquid chromatography (HPLC) is a chromatographic technique that can separate a mixture of substances, and it is used in biochemistry and analytical chemistry to identify, quantify, and purify different components of the mixture [46]. HPLC employs various types of the stationary phases, and the pump that drives the mobile phase(s) and analyte through the column and detector to provide a characteristic retention time for the analyte. The retention period of an analyte varies according to the strength of its interactions with the stationary phase, solvent(s) ratio/composition utilized, and flow rate of the mobile phase [47]. HPLC has a number of advantages, including low organic solvent utilization, minimal sample volume, quick analysis, and high chromatographic resolution [48]. Apart from conventional HPLC, other sophisticated HPLC-based techniques have been widely applied for the determination of pharmaceuticals, including antibiotics in various samples. The referred methods include liquid chromatography—mass spectrometry (LC-MS) [49,50], ultra-high performance liquid chromatography-MS/MS (UHPLC-MS/MS) [51,52,53], and liquid chromatography linked to tandem mass spectrometry (LC-MS/MS) [54,55].

### 2.2. Spectrophotometric Methods

Spectrophotometric methods are based on the creation of the complex between the pharmaceutical and the reagent [12]. The intensity of the color is used to calculate pharmaceutical concentration. The complex generated by the pharmaceutical and reagent can either be charge transfer or ion-pair in nature. The charge transfer complex, also known as the electron donor-acceptor complex, transfers a fraction of electrical charge between molecules. Coulomb attraction holds oppositely charged ions together in solution in the ion-pair complex [56]. Some antibiotics have been analyzed using spectrophotometric methods including amoxicillin [40], azithromycin [41,57], tetracycline, doxycycline [58], and cefixime trihydrate [59]. They have also been used to analyze gentamicin sulfate [60], cefadroxil, ceftazidime, cefazolin sodium, cefoperazone sodium, cefaclor, cephaprin sodium, cefotaxime sodium, and cefuroxime sodium [61].

### 2.3. Electrochemical Methods

The measurement of the current, charge, and potential is utilized in electrochemical techniques to characterize an analyte’s chemical reactivity and detect the concentration. The basic electrochemical signals that serve as analytical signals constitute current, charge, and potential [62]. These techniques include cyclic voltammetry, chronoamperometry, electro-chemical impedance spectroscopy, and potentiometry [63]. In comparison to separation and spectral methods, electrochemical methods offer practical advantages such as operation simplicity, satisfactory sensitivity, a wide linear concentration range, low instrument cost, miniaturization capability, suitability for real-time detection, and less sensitivity to matrix effects [34,64]. Due to advances in electronics and computer sciences, the electroanalysis of pharmaceutically active substances is actively involved in new study fields of various methodologies. Due to their great sensitivity and selectivity, many innovative electroanalytical techniques have been effectively employed for trace analyses of essential pharmaceutically active substances [65]. The electrochemical analysis of active pharmaceuticals is based on redox processes that occur via electron transfer channels [66]. Electrochemical methods have been used for the analysis of antibiotics such as clarithromycin and azithromycin [67], diclofenac [68], and cefixime [69].

### 2.4. Methods Studied for Analysis of Ceftriaxone in Aquatic and Biological Samples

Ceftriaxone levels have been estimated using a variety of techniques including HPLC, high performance thin layer chromatography, capillary electrophoresis, and spectrophotometry [37,70]. Literature shows a higher proportion of the usage of HPLC in the analysis of ceftriaxone in the aqueous and biological samples [14]. The methods studied for the analysis of ceftriaxone include high-performance liquid chromatography coupled with mass spectrometry detection (HPLC-MS) [14,71,72], high-performance liquid chromatography with detection by ultraviolet (HPLC-UV) [14,73,74,75,76], and high-performance liquid chromatography coupled with sequential mass spectrometry (HPLC-MS/MS) [14,77,78,79,80,81]. The referred methods studied for the analysis of ceftriaxone also include ultra-performance liquid chromatography with detection by ultraviolet (UPLC-UV) [14,82] and ultra-performance liquid chromatography coupled with sequential mass spectrometry (UPLC-MS/MS) [49,83,84]. The linear range, limit of detection, and recovery of these methods are given in Table 2.

Absorption spectroscopy methods such as ultraviolet (UV) [14,86,87,88,89,90], infrared spectroscopy [14,88,90,91,92], spectrofluorimetry [14,93], microbiological methods [14,94,95], and capillary zone electrophoresis [96] have also been used for the analysis of ceftriaxone. When used as an identification technique, UV has limited selectivity because multiple compounds may have the same or similar spectra. As a result, this technique is typically supplemented with additional spectroscopic techniques such as IR for positive analyte confirmation [35].

## 3. Methods Used for Removal of Antibiotics from Wastewater

The selection of the method for wastewater treatment depends on the characteristics of the wastewater and features such as costs, feasibility, efficiency, practicability, dependability, impact on the environment, sludge production, difficulty in operation, pretreatment demands, and the formation of potentially dangerous by-products which characterize the relevant method [97]. The potential of various techniques to remove antibiotics from wastewater systems has been investigated. Among those techniques are constructed wetlands, biological treatment, advanced oxidation processes (AOPs), and membrane technology [23].

### 3.1. Constructed Wetland

A constructed wetland (CW) wastewater treatment system utilizes the combined influence of microbes, plants, and soil to remove the pollutants from wastewater. The wastewater is treated through microbial decomposition, adsorption, plant uptake, ion exchange, co-precipitation, and filtration [98]. The suitability of CWs for the elimination of some pharmaceuticals and personal care products (PPCPs) has recently been studied [26].

Diclofenac, ibuprofen, naproxen, ketoprofen, salicylic acid, triclosan, sulfamethoxazole, carbamazepine, clofibric acid, atenolol, and caffeine are some of the pharmaceuticals that have been investigated in constructed wetlands [99,100]. The average removing efficiencies of constructed wetlands are 93% (monensin), 89% (ofloxacin), 87% (oxytetracycline), 83% (sulfapyridine), 80% (caffeine), 79% (salicylic acid), 72% (atenolol), 72% (furosemide), 69% (doxycycline), 68% (codeine), 67% (diltiazem), 64% (acetaminophen), 62% (naproxen), 57% (ibuprofen), 56% (metoprolol), and 51% (sulfadiazine) to some studied pharmaceuticals [101]. Several studies have shown that physico-chemical decomposition, photodegradation, adsorption by wetland soil and plants, and biodegradation (microbial activity) comprise the mechanisms used to remove antibiotics from wastewater in CWs [67,68]. Antibiotics can accumulate in plants by water transport and passive absorption and high quantities of antibiotics in water or soil can be harmful to plant development and metabolic activity [102]. Since there are very few informative publications on the decontamination of antibiotics using CWs, this area of research could benefit from combined support from other disciplines, primarily soil science, botany, environmental chemistry, and chemical engineering [103].

### 3.2. Biological Treatment

The microorganisms utilize organic compounds and nutrients to gain energy and build the blocks for their growth in biological treatment methods. Despite the presence of high density and diverse consortium of microorganisms in activated sludge, antibiotics cannot be completely removed in biological treatment methods [104]. Some reasons for the incomplete removal of antibiotics in biological methods include relatively low concentration of antibiotics in the wastewater, which leads to a lack of enzymes responsible for antibiotic biodegradation and inhibitory or toxic properties of antibiotics that can stop the microorganism activity responsible for antibiotic biodegradation, antibiotic properties, and operation conditions [18]. Different biological treatment methods have been investigated in relation to the removal of antibiotics from wastewater. For instance, using a biological aerated filter system (BAF), 89–91% of nine antibiotics were removed from swine wastewater. Those antibiotics include oxytetracycline, leucomycin, lincomycin, ofloxacin, trimethoprim, norfloxacin, sulfamonomethoxine, sulfamethazine, and sulfachloropyridazine [105]. Using anaerobic digestion, 65% tetracyclines and 85% of quinolones were removed from swine wastewater after 16d hydraulic retention time (HRT) [106].

Another study indicated that the lab-scale intermittently aerated sequencing batch reactor (IASBR) was applied to treat anaerobically digested swine wastewater. The results from the referred study show that 87.9% tetracyclines were removed, and 96.2% sulfonamides were removed at about 3–5 d HRT [107]. The elimination of antibiotics using the sequencing-batch membrane bioreactor (SMBR) was investigated for the treatment of swine wastewater. Nine antibiotics, which were divided into sulfonamides, tetracyclines, and fluoroquinolones, and three categories of frequently used veterinary antibiotics were investigated. The results demonstrated that SMBR effectively removed sulfonamides and tetracyclines (90%), whereas fluoroquinolones were removed less effectively (70%) [108]. Many antibiotics have been identified in the literature as being resistant to biodegradation. While some antibiotics can be partially decomposed, the majority of antibiotics including ciprofloxacin, metronidazole, ceftriaxone, ofloxacin, and trimethoprim are not biodegradable [73,74]. More research is needed to understand the factors affecting the process and possibility of improving the degradation of pharmaceuticals.

### 3.3. Advanced Oxidation Processes (AOP)

AOPs comprise water and wastewater treatment technologies that use powerful oxidizing agents such as hydroxyl radical (OH•), ozone (O_3_), chloride (Cl^−^), and superoxide radical (O_2_^−^) [109]. The generated species react with the medium’s organic molecules [110] to start a series of oxidation reactions until all of the components have been mineralized to CO_2_ and H_2_O [111]. AOP methods can be divided according to the source of OH• production with UV–hydrogen peroxide processes, with Fenton and photo-Fenton, ozone-based processes, photocatalysis, and sonolysis being the most common [112]. Such methods have proven to be effective at removing a wide range of contaminants in general and antibiotics in particular [110]. Electrochemical oxidation was used to study the removal of tetracycline (TC) antibiotics from the livestock wastewater. The electrochemical treatment of the TC in aqueous solutions for 6 h with a Ti/IrO_2_ anode and Na_2_SO_4_ electrolyte resulted in concentrations decreasing from 100 mgL^−1^ to less than 0.6 mgL^−1^ [113].

With sinusoidal alternating electro-Fenton (SAEF), the removal efficiency and the mechanism of TC degradation were studied. According to the findings, the removal rates of TC were 94.87% in optimal conditions [114]. A study was done to examine the efficacy of three AOPs for removing antibiotics from wastewater: ozonation, photo-Fenton process, and heterogeneous photocatalytic process with a TiO_2_ semiconductor. The ozonation process was discovered to be effective at removing all types of antibiotics [115]. The majority of the literature to date, however, has been devoted to bench- or pilot-scale experiments. The use of AOPs on a large scale is still a work in progress. The high operational cost of AOPs, especially when compared to the conventional methods that are routinely used today, is likely to be the greatest challenge for the development of AOPs on an industrial scale [110]. Further research is needed to address the challenges associated with AOPs in attempt to make the processes affordable and useful in the real wastewater treatments.

### 3.4. Membrane Technology

A membrane is described as a thin layer, film, or sheet that serves as a specific barrier between two phases which may be vapor, gas, or liquid. To put it in another way, a membrane is the boundary between two adjacent phases that function as a selective barrier to control the movement of species between the two compartments. Membrane technology includes the associated engineering and scientific techniques for transporting or excluding the parts, species, or substances from membranes [116]. Ultrafiltration (UF), electrodialysis (ED), membrane distillation (MD), microfiltration (MF), nanofiltration (NF), particle filtration (PF), pervaporation (PV), reverse osmosis (RO), and membrane bioreactor (MBR) are just a few of the membrane-based technologies that have been developed based on the impurities that need to be removed and the method of application [117,118].

Various membrane technologies have been evaluated for pharmaceutical removal at both the pilot and full-scale levels [119]. The membrane technology is preferred due to significant reductions in equipment size, energy requirements, and low capital costs. It has the potential to close the economic and sustainability gap with low or no chemical usage, environmental friendliness, and ease of access for many [120]. A few studies have investigated the removal of antibiotics from wastewater using membrane technology. For instance, one study on wastewater treatment indicate that the rate of antibiotic removal was 87% when UV/ozone and nanofiltration were used [121]. The combination of nanofiltration and reverse osmosis technologies was utilized to treat swine wastewater and efficiently removed various antibiotic resistant genes [122]. As a conclusion, additional research on the use of membrane technology to remove antibiotics from wastewater should be done.

## 4. Methods Studied for Removal of Ceftriaxone from Water and Wastewater

The techniques studied regarding the removal of ceftriaxone from aqueous systems include photochemical degradation, ion ex-change, chemical oxidation, biological treatment, and adsorption [123]. Table 3 summarizes some of the studies on the methods for the removal of ceftriaxone from wastewater.

## 5. Conclusions

Despite the fact that ceftriaxone is one of the most commonly prescribed antibiotics in health facilities, this review demonstrates that there is little information on its occurrence in the environmental samples. Due to potential consequences of their presence in the environment, it is necessary to examine and monitor their presence. The majority of studies on the strategies for the degradation or removal of ceftriaxone from various samples are based on AOPs. The most significant disadvantage of the AOP methods is their expense, which comprise the operating and maintenance costs associated with the system’s needs for energy and chemical reagents. Despite the evidence that some approaches such as biological procedures cannot remove ceftriaxone, further research is needed to study the possibilities of other alternatives such as constructed wetland systems. The majority of the reviewed studies were conducted on a small scale in the laboratory under controlled environments. Alternative research is required to determine the feasibility and effectiveness of the techniques for degrading ceftriaxone in wastewater by involving the complex mixtures of contaminants and variations in weather conditions.

## Figures and Tables

**Table 1:** Characteristics of ceftriaxone sodium [16].

Characteristics	Value
Physical properties	Crystalline white powder
Solubility	Soluble in water (app. 40 g/100 mL at 25 °C)
Ionization constants (pKa)	4.1 (enolic OH), 3.2 (NH_3_^+^) and 3 (COOH)
Route of elimination	By glomerular filtration, ceftriaxone is eliminated unaltered in the urine. Bile excretes around 35–45% of a given dosage of ceftriaxone.

**Table 2:** Comparison of chromatographic methods used for determination of ceftriaxone [85].

Type of Technique	Sample Used	Limit of Detection (µgL^−1^)	Range of Linearity (µgL^−1^)	% Recovery
HPLC-UV	Hospital wastewater	2.0	5.0–600	152.38
HPLC-MS/MS	Human plasma		3.0–300	87.35
HPLC	Sterile powder for injection		20–150	99.42
HPLC	Human urine	0.05	0.24–250	97.73–100.7
RP-HPLC	Pharmaceutical formulation	0.51–1.54	2.5–25	˃98.1

**Table 3:** Methods for removal of ceftriaxone from aqueous solution.

Method	Results	Reference
Chemical oxidation	Degradation occurs through Type I and Type II mechanisms.	[124]
UVC/H_2_O_2_ and UVC	At a solution pH of 5 and an H_2_O_2_ concentration of 10 mg/L, the most ceftriaxone degradation was observed. Pseudo-first- and second-order kinetics models with reaction rate constants of 0.0165 and 0.0012 min^−1^, respectively, better represent UVC/H_2_O_2_ and UVC processes.	[11]
O_3_/UV/Fe_3_O_4_@TiO_2_	Maximum ceftriaxone removal 92.40% Organic carbon reduction 72.5% Optimal conditions, time: 30 min, photocatalyst dosage: 2 g/L, pH: 9, initial ceftriaxone concentration: 10 mg/L, and ozone dosage: 0.2 g/h)	[125]
Immobilized TiO_2_ and ZnO	Results revealed that photodegradation using UV/TiO_2_ process was more effective than photodegradation using the UV/ZnO process. Ceftriaxone photodegradation followed pseudo-first-order kinetics in both systems.	[126]
Electrochemical in aqueous solutions containing sodium halides	Ceftriaxone gradually decomposes, but not fully, in the presence of fluoride ions in about 60 min without yielding a reaction product. The electro (degradation/transformation) of ceftriaxone is practically complete in 10 and 5 min with completion of the electro-transformation reaction, which take 60 and 30 min, respectively. Ceftriaxone and the iodide ions formed instantaneous interactions.	[127]
Heterogeneous catalytic AOP γ-Fe_2_O_3_ encapsulated NaY zeolites solid adsorbent	initial concentration of 20 mg/L, catalyst 1.17 g/L, H_2_O_2_ 30 mM, and UV light, ceftriaxone may be effectively removed within 90 min at pH 4.0. The adsorption mechanism was investigated using the kinetic and isotherm model, and the results demonstrate that the model and data are in good agreement.	[128]

## Data Availability

Not applicable.
